# Effect of short-term exposure to air pollution on daily cardio- and cerebrovascular hospitalisations in areas with a low level of air pollution

**DOI:** 10.1007/s11356-023-29544-z

**Published:** 2023-09-05

**Authors:** Md Golam Hasnain, Carlos Garcia-Esperon, Yumi Kashida Tomari, Rhonda Walker, Tarunpreet Saluja, Md Mijanur Rahman, Andrew Boyle, Christopher R. Levi, Ravi Naidu, Gabriel Filippelli, Neil J. Spratt

**Affiliations:** 1 College of Health, Medicine, and Wellbeing, The University of Newcastle, University Drive, Callaghan, New South Wales 2308 Australia; 2 John Hunter Hospital, Hunter New England Local Health District, Lookout Road, New Lambton Heights, New South Wales 2305 Australia; 3 Hunter Medical Research Institute, Lookout Road, New Lambton Heights, New South Wales 2305 Australia; 4 The Daffodil Centre, The University of Sydney, A Joint Venture with Cancer Council NSW, Sydney, Australia; 5 Global Centre for Environmental Remediation, College of Engineering Science and Environment, The University of Newcastle, University Drive, Callaghan, New South Wales 2308 Australia; 6 CRC for Contamination Assessment and Remediation of the Environment (crcCARE), The University of Newcastle, University Drive, Callaghan, New South Wales 2308 Australia; 7 Department of Earth Sciences, Indiana University, Indianapolis, IN 46202 USA

**Keywords:** Low air pollution, Short-term exposure, Cardiovascular disease, Cerebrovascular disease, Hospitalization, Australia

## Abstract

Exposure to air pollution is associated with increased cardio- and cerebrovascular diseases. However, the evidence regarding the short-term effect of air pollution on cardio- and cerebrovascular hospitalisations in areas with relatively low air pollution levels is limited. This study aims to examine the effect of short-term exposure to different air pollutants on hospital admissions due to cardio- and cerebrovascular diseases in rural and regional Australia with low air pollution. The study was conducted in five local Government areas of Hunter New England Local Health District (HNE-LHD). Hospitalisation data from January 2018 to February 2020 (820 days) were accessed from the HNE-LHD admitted patients’ dataset. Poisson regression model was used to examine the association between the exposure (air pollutants) and outcome variables (hospitalisation due to cardio- and cerebrovascular disease). The concentrations of gaseous air pollutants, Sulphur Dioxide (SO_2_), Nitrogen Dioxide (NO_2_), Ozone (O_3_), Carbon Monoxide (CO), and Ammonia (NH_3_) were below national benchmark concentrations for every day of the study period. In single pollutant models, SO_2_ and NO_2_ significantly increased the daily number of cardio- and cerebrovascular hospitalisations. The highest cumulative effect for SO_2_ was observed across lag 0–3 days (Incidence Rate Ratio, IRR: 1.77; 95% Confidence Interval, CI: 1.18–2.65; *p*-value: 0.01), and for NO_2_, it was across lag 0–2 days (IRR: 1.13; 95% CI: 1.02–1.25; *p*-value: 0.02). In contrast, higher O_3_ was associated with decreased cardio- and cerebrovascular hospitalisations, with the largest effect observed at lag 0 (IRR: 0.94; 95% CI: 0.89–0.98; *p*-value: 0.02). In the multi-pollutant model, the effect of NO_2_ remained significant at lag 0 and corresponded to a 21% increase in cardio- and cerebrovascular hospitalisation (95% CI: 1–44%; *p*-value = 0.04). Thus, the study revealed that gaseous air pollutants, specifically NO_2_, were positively related to increased cardio- and cerebrovascular hospitalisations, even at concentrations below the national standards.

**Supplementary Information:**

The online version contains supplementary material available at 10.1007/s11356-023-29544-z.

## Background

Air pollution is deemed one of our era’s greatest man-made curses because it influences climate change and increases multiple health hazards (World Health Organization, WHO, 2016). Globally, 4.2 million deaths and 103.1 million lost years of healthy life are attributable to air pollution, most of which are secondary to cardio- and cerebrovascular diseases (Bourdrel et al. 2017; Liu et al. 2020; Mustafic et al. 2012). Short- and long-term exposure to ambient air pollution can cause increased cardio- and cerebrovascular hospitalisation up to twofold. Published systematic reviews have reported a 15 to 19% increase in stroke-related hospital admission or mortality and a two to three times increase in heart-failure-related hospitalisation and death (Shah et al. 2013; Shah et al. 2015).

Over the last 100 years, there has been a dramatic increase in air pollution. However, studies of the effect of air pollution on cardio- and cerebrovascular diseases have been done in high pollution areas. A recently published umbrella review of systematic reviews and meta-analysis (de Bont et al. 2022) showed substantial evidence of an association between exposure to air pollution and an elevated risk of cardio- and cerebrovascular diseases. Most of the data in the 56 reviews and meta-analyses included in the research came from nations with moderate to high levels of air pollution. This data is very important to demonstrate the harms that can be caused by air pollution; however, to determine the minimum safe levels of such pollutants, we need studies from areas with very low basal levels of pollution such as Australia, in order to determine the levels at which there is no evidence of health-related harms. Therefore, we conducted this research to answer the following research question “what is the short-term effect of ambient air pollution on daily number of cardio- and cerebrovascular hospital admissions in areas with low level of air pollution?”

Air pollution comes in various forms and previous research findings identified that the most common and harmful air pollutants are particulate matter (PM) (including those under 2.5 μm diameter, or PM_2.5_, and over 2.5 and under 10 μm diameter, or PM_10_), sulphur dioxide (SO_2_), nitrogen dioxide (NO_2_), carbon monoxide (CO), ammonia (NH_3_) and ozone (O_3_) (New South Wales, NSW, Health, 2013).

In Australia, air quality is among the best in the world, ranked as the 10^th^ cleanest country in 2019 (IQAir Air Quality Report, 2019). However, despite having a low average level of air pollution, serious health events may still be significant (Bourdrel et al. 2017). Air pollutants were directly linked to 7% of the total coronary heart disease, 4% of stroke, 3% of chronic obstructive pulmonary disease, 2% of lower respiratory infections and 1% of lung cancer burden in Australia (NSW Health, 2013). However, most reported results are from major metropolitan cities, and evidence from rural and regional areas is limited.

The region, Hunter New England, is predominantly rural, with one regional metropolitan area and a population of 920,370 (NSW Ministry of Health, 2016). It is a major coal-mining area; however, the air quality of the Hunter region is generally reasonable. Over the past 5 years, 2017–2021, gaseous air pollutants were below national benchmark concentrations on all days. Particulate matter readings were also below national benchmark concentrations on approximately 95% of the days, except for 2019, which was heavily affected by significant bushfires (Air Quality Monitoring Reports, 2017-2021).

Therefore, to answer the above-mentioned research question, this study aimed to examine the effects of short-term air pollution on hospitalisations for cardio- and cerebrovascular diseases in five Local Government Areas (LGAs) of Hunter New England Local Health District (HNE-LHD). It encompasses one urban and several regional communities with a historical background of low levels of air pollution.

## Methods

### Study area and population

**Fig. 1:**
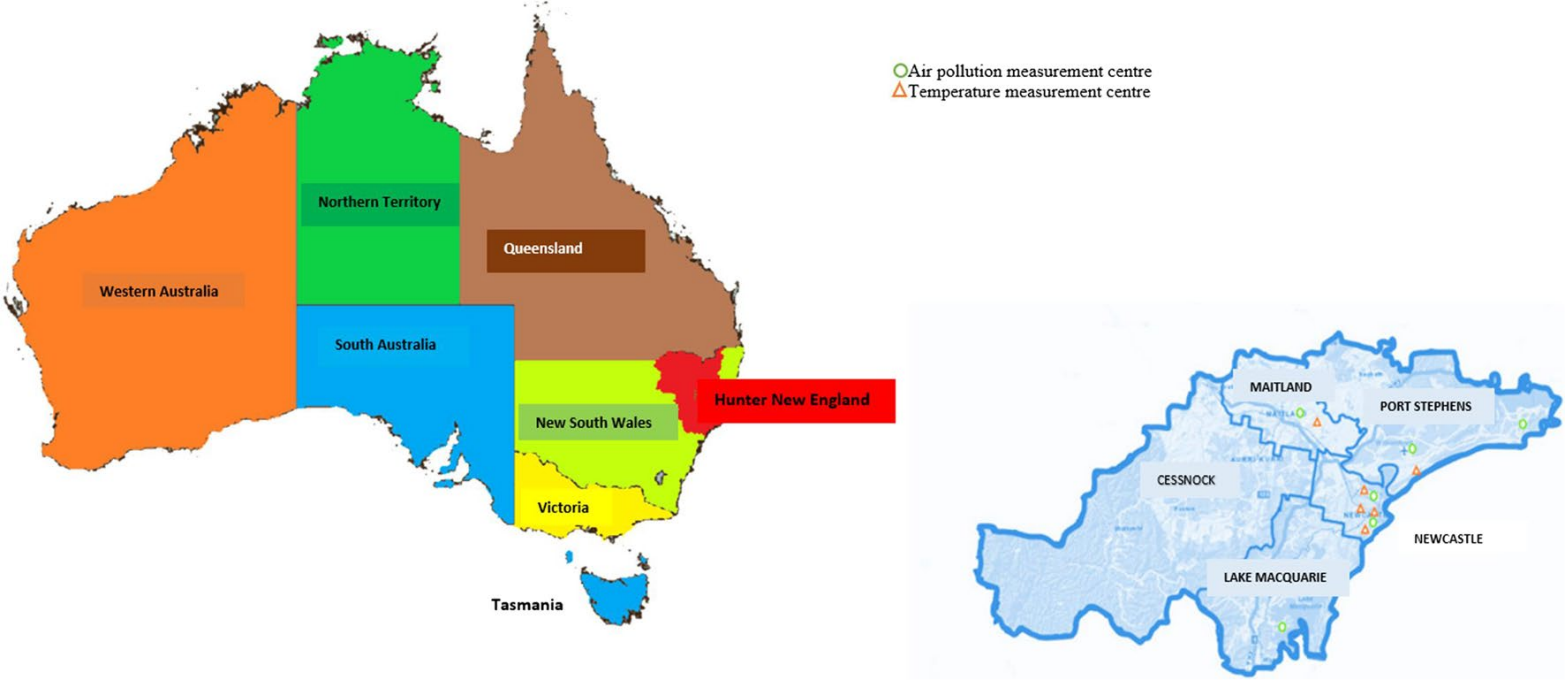
Diagram showing the distribution of air pollutant and weather-measurement centres across the selected LGAs (Mapping important agricultural lands in the lower hunter region of NSW and Primary Health Network Plan (n.d.): About us)

The study was conducted in HNE-LHD, encompassing an area of 131,785 km^2^ with 22 LGAs (Hunter New England, NSW Health n.d.). However, this study focused on five LGAs of HNE-LHD: Newcastle, Lake Macquarie, Port Stephens, Maitland, and Cessnock; Fig. 1. These areas were chosen as they have urban and rural areas and have the highest population density. We have a very limited number of air pollution monitoring stations in areas of the HNE-LHD outside the included areas. Inclusion of areas with very low population density and small number of monitoring stations would likely result in spatial variation, exposure misclassification, and an under- or overestimation of the true health effects. According to the national census of 2016, these five LGAs had a population of 570,986, with the highest population density in Newcastle LGA, 860.6 persons per km^2^, followed by 311.9 at Lake Macquarie, 201.9 at Maitland, 82.8 at Port Stephens and 28.9 at Cessnock. Collectively, these five LGAs account for 62% of the HNE-LHD population within 3% of the total land area (NSW Ministry of Health, 2016).

### Outcome variable: daily hospitalisation

The daily number of hospitalisations due to cardio- and cerebrovascular diseases was considered the outcome variable. The hospitalisation data from January 2018 to February 2020 were accessed from the HNE-LHD Cardiac and Stroke Outcomes database, which collates information on discharges from all publicly funded hospitals within HNE-LHD. All the cases were coded according to the World Health Organisation’s (WHO’s) International Classification of Diseases, 10th Revision (ICD-10). Cardiovascular diseases (a group of disorders of heart and blood vessels) were coded as I20-I52 and I70-I99, ischaemic heart diseases (conditions caused by narrowed heart arteries, coronary arteries, that supply blood to the heart muscle; also known as coronary heart or coronary artery disease) as I20-I25, cerebrovascular diseases (a group of disorders of blood vessels supplying the brain and its membrane) as I60-I69, haemorrhagic stroke (a condition caused by ruptured brain blood vessels) as I60-I62 and ischaemic stroke (a condition caused by the blockage of the brain blood vessels) as I63 (ICD-10 version, WHO, 2016). Daily counts (cases) of the number of patients with an address within the five selected LGAs with any diagnosis identified on discharge (principal diagnosis only) according to the above-mentioned ICD-10 code were obtained. The admission date was the incidence date for analysis and considered the day of pollutant exposure.

### Exposure variable: concentration of air pollutants

Air pollutants data were collected from six air pollution measuring stations within the five selected LGAs: Newcastle, Beresfield, Wallsend, Carrington, Mayfield, and Stockton, Fig. 1. Data on seven air pollutant (PM_10,_ PM_2.5,_ SO_2,_ NO_2,_ O_3,_ CO, and NH_3_) were collected from the six stations. The daily mean concentration of each air pollutant was considered the exposure variables collected from the NSW Government Planning, Industry and Environment website (NSW Government Air Quality Data Services, 2020). Data on PM_10,_ PM_2.5,_ SO_2,_ and NO_2_ were available in all six selected stations, wherein O_3_ was available in three stations and CO and NH_3_ in only one station. The daily maximum temperature and total rainfall data were also collected from the six weather stations, Fig. 1, selected within the selected LGAs using the Australian Government Bureau of Meteorology website (Climate Data Online, Australian Government: Bureau of Meteorology, 2020).

### Measuring the association between air pollution and diseases

After accumulating data on air pollutants and hospital admissions, we used regression models to determine the impact of each pollutant on the daily number of cardio- and cerebrovascular hospitalisations. Taking into account the potential delayed or cumulative effects of the pollutants, we also measure the effects of lagged days. We began with single pollutant models and, based on the outcomes of these models, moved on to bi- and multi-pollutant models.

### Statistical analysis

All analyses were performed with Stata Statistical Software, version 15 (Stata Corp, College Station, TX, USA). Data were summarised using descriptive statistics: frequency and percentages for dichotomous variables and mean and standard deviation or median and Inter Quartile Range (IQR) for continuous variables. Poisson regression models were used to measure the effect of each air pollutant on cardio- and cerebrovascular hospitalisation. We conducted the goodness-of-fit chi-squared test to check whether the data fits with the model reasonably or not. We also compared the residual plots (checked the overdispersion) with negative binomial logistic regression models to reach on the final decision in selecting models. Each regression model was adjusted for daily maximum temperature, daily total rainfall, and seasonal pattern. Considering the possible delayed and or cumulative effects of air pollution, we created a cross-basis matrix for air pollutants within the distributed lag linear model framework. The selection of lag days was justified by the information criteria: Schwarz’s Bayesian information criterion (SBIC), Akaike’s information criterion (AIC), and the Hannan and Quinn Information Criterion (HQIC). Initially, the lag length was set as 10 days, and the results showed that AIC supported the selection of maximum lag days to be five, while the other two information criteria indicated considering 1 day lag is preferable. (Supplementary Table 1). However, we considered 5 days lag (larger lag days) to prevent the possibilities of increased variances and spurious estimates (Kim et al. 2019) for the later analysis. Incidence Rate Ratio (IRR) and 95% Confidence Intervals (95% CI) were estimated from the model. Pollutants with significant effect were included in the bi- and multi-pollutant models. The effects were then estimated using bi- and multi-pollutant models. Single pollutant models were used to estimate the impacts, and then multi-pollutant models were used to account for the possible confounding effects of multiple pollutants. A *p*-value < 0.05 is considered significant.

## Results

Between January 2018 and February 2020, 4861 admissions were recorded with cardio- and cerebrovascular diseases. Of these, 3344 (68.79%) admissions were for patients with cardiovascular diseases, the majority, 2678 (80.08%), with ischaemic heart diseases. A total of 1517 (31.21%) admissions were for cerebrovascular diseases, of which 999 (65.85%) were for ischaemic stroke and 351 (23.18%) for haemorrhagic stroke.

Of the 4861 patients, 2057 (42.32%) were male, 218 (5.23%) identified as Aboriginal or Torres Strait Islander, the median age was 79 (59–81) years, and 3190 (65.62%) were older than 65 years. The median daily cardio- and cerebrovascular hospitalisation was 6 (IQR: 5–8). For cardiovascular hospitalisations, the median value was 4 (IQR: 3–5), and for cerebrovascular diseases, the value was 2 (IQR: 1–3).

Over the period studied, the 24 h mean concentrations of gaseous pollutants were: SO2 - 0.18 ± 0.11 parts per hundred million (pphm), NO_2_ - 0.73 ± 0.45 pphm_,_ O_3_, and NH_3_ were, 1.79 ± 0.64, and 1.19 ± 1.42, respectively. The 24 h mean concentration of CO was 0.25 ± 0.11 parts per million (ppm). The 24 h mean concentrations of PM10 and PM_2.5_ were 29.09 ± 16.21 and 10.05 ± 8.15 μg/m^3^, respectively. During the study period, the concentrations of gaseous air pollutants were below the national benchmark for every day. However, concentrations for the particulate matter, PM_10_, and PM_2.5_ were above the benchmark for 52 (6.34%) days and 28 (3.41%) days, respectively. A detailed description of the basic characteristics of disease admissions, meteorological data, and air pollution data are presented in Supplementary Table 2. The correlation between air pollution and meteorological factors is shown in Supplementary Table 3.

### Single pollutant models

#### Cardio- and cerebrovascular diseases

**Fig. 2:**
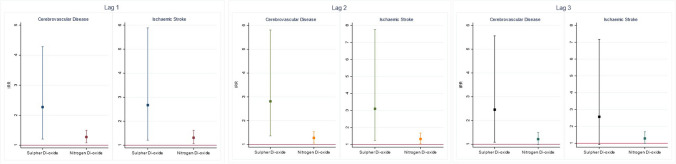
IRRs (95% CI) of cerebrovascular disease and ischaemic stroke hospitalisation for each unit increase in ambient SO_2_ and NO_2_in the single pollutant model

SO_2_ and NO_2_ showed positive associations between the daily concentrations and the number of cardio- and cerebrovascular hospitalisations, Fig. 2. The highest cumulative effect for SO_2_ was observed across lag 0–3 days (IRR: 1.77; 95% CI: 1.18–2.65; *p*-value: 0.01), and for NO_2,_ it was across lag 0–2 days (IRR: 1.13; 95% CI: 1.02–1.25; *p*-value: 0.02). On the other hand, a negative association was observed between the daily concentration of O_3_ and daily hospital admissions due to cardio- and cerebrovascular diseases, Supplementary Table 4. The highest cumulative effect was observed in O_3_ at lag 0 (IRR: 0.94; 95% CI: 0.89–0.98; *p*-value: 0.02). No significant association was observed between overall daily cardio- and cerebrovascular hospitalisations and concentrations of other air pollutants (CO, NH_3_, PM_10_ and PM_2.5_), Supplementary Table 4 and Supplementary Table 5.

#### Cerebrovascular diseases

Similarly, the effect of SO_2_ and NO_2_ remained the same with cerebrovascular-only hospitalisations, Fig. 2. The maximum effect was observed across lag 0–2 (SO_2_ = IRR: 2.81; 95% CI: 1.36–5.81; *p*-value: 0.01 and NO_2_ = IRR: 1.28; 95% CI: 1.06–1.54; *p*-value: 0.01). No association was observed with other pollutants (O_3_, CO, NH_3_, PM_10_ and PM_2.5_), Supplementary Table 4 and Supplementary Table 5. A further sub-group analysis revealed that the effect continued with the daily number of ischemic stroke admissions and the haemorrhagic stroke, although the effects were statistically significant only for ischemic stroke. The highest IRR for SO_2_ was 3.10 (95% CI: 1.24–7.76; *p*-value: 0.015) and for NO_2_ was 1.33 (95% CI: 1.05–1.68; *p*-value: 0.019) (Fig. 2).

#### Cardiovascular diseases

No association between cardiovascular-only hospitalisations and daily air pollutants concentrations was observed. Further analysis limited to ischaemic heart disease produced similar results (Supplementary Table 4 and Supplementary Table 5).

### Bi- and multi-pollutant models

Results from the bi-pollutant models also showed on Supplementary Table 6. Finally, in the multi-pollutant model where SO_2,_ NO_2,_ and O_3_ were included, the effect estimates of SO_2_ and O_3_ dropped compared to their effect on the single-pollutant models, Table 1. As a result, only the effect of NO_2_ concentrations remained significant and accounted for a 21% increase in daily cardio- and cerebrovascular hospitalisations (95% CI: 1–44%; *p*-value: 0.040).

**Table 1:** Cardio- and cerebrovascular hospitalisations’ IRRs (95% CI) for every unit increase in ambient SO_2,_ NO_2,_ and O_3_ in the multi-pollutant model, across lag 0–5 days

Outcome	Lag	IRR (95% CI); *p*-value		
		SO_2_	NO_2_	O_3_
Cardio- and cerebrovascular disease	0	1.04 (0.75–1.43); 0.83	1.08 (0.98–1.20); 0.12	0.96 (0.91–1.00); 0.07
	1	1.13 (0.75–1.68); 0.56	1.08 (0.96–1.21); 0.20	0.96 (0.91–1.01); 0.14
	2	1.40 (0.88–2.20); 0.15	1.05 (0.93–1.19); 0.42	0.97 (0.91–1.03); 0.27
	3	1.57 (0.96–2.57); 0.07	1.03 (0.90–1.18); 0.67	0.97 (0.91–1.03); 0.37
	4	1.62 (0.96–2.75); 0.07	0.98 (0.85–1.13); 0.80	0.97 (0.91–1.04); 0.39
	5	1.48 (0.86–2.56); 0.16	0.99 (0.86–1.14); 0.86	0.97 (0.91–1.04); 0.35
Cerebrovascular disease	0	1.10 (0.60–2.02); 0.76	1.21 (1.01–1.44); 0.04*	0.95 (0.87–1.04); 0.28
	1	1.57 (0.72–3.43); 0.26	1.16 (0.94–1.43); 0.16	0.97 (0.87–1.07); 0.51
	2	2.11 (0.86–5.17); 0.10	1.12 (0.89–1.42); 0.33	0.98 (0.88–1.10); 0.69
	3	1.98 (0.71–5.46); 0.19	1.07 (0.82–1.40); 0.60	0.97 (0.85–1.09); 0.59
	4	1.90 (0.65–5.58); 0.24	1.05 (0.79–1.39); 0.76	0.97 (0.85–1.10); 0.63
	5	1.76 (0.58–5.39); 0.32	1.09 (0.81–1.47); 0.58	0.97 (0.85–1.11); 0.67
Ischaemic stroke	0	1.38 (0.67–2.84); 0.39	1.20 (0.96–1.52); 0.11	0.99 (0.88–1.01); 0.87
	1	1.92 (0.73–5.10); 0.19	1.21 (0.93–1.58); 0.16	1.04 (0.91–1.18); 0.60
	2	2.33 (0.77–7.07); 0.13	1.21 (0.90–1.62); 0.21	1.06 (0.91–1.23); 0.44
	3	1.93 (0.57–6.59); 0.29	1.20 (0.87–1.67); 0.27	1.05 (0.90–1.23); 0.54
	4	1.66 (0.46–6.04); 0.44	1.17 (0.82–1.67); 0.38	1.05 (0.89–1.24); 0.55
	5	1.35 (0.36–5.08); 0.66	1.26 (0.88–1.82); 0.21	1.06 (0.90–1.25); 0.49

*p-value <0.05 considered significant

## Discussion

We found that short-term exposure to ambient gaseous pollutants, particularly NO_2_, was associated with increased cerebrovascular hospitalisations. These findings are remarkable because this effect was seen despite all the gaseous air pollutants being within the national benchmark of maximum permissible concentrations for every day of the study period. We saw no association between particulate matter, considered the most common and harmful air pollutant, and the daily number of cardio- and cerebrovascular hospitalisations. This may be related to the overall low exposure, although particulate concentrations did exceed national benchmarks on 3–6% of days.

The effect of NO_2_ in this study on the daily total of cerebrovascular hospitalisations was similar to findings from earlier studies, primarily from regions with high to moderate levels of air pollution (de Bont et al., 2022), supporting the veracity of these findings. In addition, we have tracked most of the pollutants known to be associated with cardio- and cerebrovascular disease, and included them in multipollutant models where appropriate, to minimise the risk of confounding. NO_2_ is well known for its effect on cardio- and cerebrovascular diseases. A recent systematic review also reported NO_2_ as a possible pollutant in increasing the frequency of daily hospital admissions for stroke, the most frequent type of cerebrovascular disease (Niu et al. 2021). Multiple very large sample size studies from Chinese cities (with high pollution levels) were included in this meta-analysis, and no subgroup analysis to investigate the influence of baseline pollution levels was undertaken. Thus, our research provides an important context for understanding the impact of even brief exposure to ambient air pollutants within a population with low baseline pollutant exposure.

In single pollutant models, we found that both NO_2_ and SO_2_ concentrations were positively associated with increased hospital admissions due to cerebrovascular diseases and, more specifically, ischaemic stroke, the most common form of cerebrovascular disease. Several studies and systematic reviews have established and validated the effect of short-term ambient exposure to both NO_2_ and SO_2_. The most recently published systematic review and meta-analysis identified a 13% increased stroke hospitalisation for SO_2_ and 23% for NO_2_ (Niu et al. 2021). However, this study was not able to separate effects on haemorrhagic and ischaemic stroke, since many of the included studies did not distinguish between the two. However, ischaemic stroke, being more common, is likely to be the major driver of results. The results from single pollutant modelling are in accordance with our findings. Effects for both NO_2_ and SO_2_ were similar, and there were consistent trends for cerebrovascular diseases, with similar lag durations, for both pollutants. This significant effect of pollutants in increasing the number of hospital admissions, despite pollutant concentrations within the recommended daily limits, highlights the need for further evaluation of the appropriateness of the current recommended national standards.

In our study, although the single pollutant model results aligned with previous studies, the effect did not persist in the multi-pollutant model. However, most of the studies included in the review were conducted in areas with high pollution levels. The pollution level of our study area is relatively low compared to the regions evaluated in other studies. The low air pollutant level might cause non-persistent findings of the single pollutant models. In addition, the effect of O_3_ was only evident with overall cardio- and cerebrovascular hospitalisation in the single pollutant model. The results from recently published literature evaluating the effect of O_3_ on cardio- and cerebrovascular disease are mixed (Lisabeth et al. 2008; Mechtouff et al. 2012; Montesor-Lopez et al. 2016; Yu et al. 2017). However, a multicentre clinical study of older healthy subjects revealed that low levels of ozone exposure caused no effects on systemic inflammation, oxidative stress or pre-thrombotic state. Exposure to pollutants triggers systemic inflammation, oxidative stress and platelet activation and thus increases the chances of having peripheral thrombosis and blood clotting as well as stroke and cerebrovascular diseases (Delfino et al. 2008; Mills et al. 2007). In addition, the air pollutant monitoring stations included in this study do not have any data on maximum 8-h average which is a more prevalent index compared with 24-h average. Therefore, the effect of O_3_ in this population still needs to be further assessed.

The effect of other gaseous pollutants, CO and NH_3_ and the particulate matters PM_10_ and PM_2.5_ was non-significant. In this regard, the current study findings do not align with previous research findings, mostly from areas with high pollution. Previous authors have shown strong associations between increased hospitalisations for cardio- and cerebrovascular diseases and the daily ambient level of PM_10_ and PM_2.5_ (Bourdrel et al. 2017; Du et al. 2016; Lee et al. 2018; Shah et al. 2015). The most plausible explanation for the apparent discrepancy is low particulate concentrations in the current study. On most days (94–97%), the concentration of particulate matter was within the World Health Organization (WHO), 2016 recommended limit (PM_10_: 50 μg/m^3^ and PM_2.5_: 25 μg/m^3^). Therefore, the low level of exposure and a small number of days with high particulate matter might not be enough to produce any measurable effect. Our failure to detect a significant effect of CO and NH_3_ concentrations should be interpreted with caution, since data regarding these pollutants were only available from one air pollutant monitoring station, which may have limited our ability to detect a true effect.

The study’s strengths include its focus on the correlation between certain air pollutants and hospitalisations for cardio- and cerebrovascular diseases in rural and regional areas of Australia. More importantly, the research was done in a region with a relatively uniform population (in terms of demographic characteristics) and ecosystem. The research area’s pollution levels were lower than the national standard benchmark and WHO recommendation, making it reasonably clean in international comparison. However, the study also includes certain caveats typical to previous research. First, it is possible that the air pollution data collected by fixed monitoring stations are not representative of the average population exposure. In addition, a straightforward daily averaging procedure could lead to measurement errors for pollutants with a correspondingly large spatial variability and varying air pollutant concentrations at six surveillance locations. The second limitation is that it is challenging to eliminate the possibility of ecological fallacy in time-series studies, and therefore we cannot provide absolute risk estimates for this investigation. Consequently, the correlations observed in this investigation cannot prove causation. The third limitation is that we were unable to account for specific confounding variables such as smoking status, hypertension, or dyslipidaemia. Finally, data were collected and coded by hospital clinical coders; consequently, we cannot rule out the possibility of diagnostic inconsistencies.

## Conclusion

The results of this research demonstrated that even when levels of gaseous air pollutants, particularly NO2, were below national standards, they were still associated with cerebrovascular hospitalizations. Our findings therefore indicated an association between air pollution and cerebrovascular disease–related hospital admissions and highlight the need of taking into account the impact of low levels of air pollution in relation to public health. However, more research is required to fully comprehend the safe limit of various pollutants in low-pollution areas, better understand the negative effects of particular pollutants on various types of cerebrovascular diseases, and further review the current national standard to lower the prevalence of cerebrovascular diseases.

### Supplementary information

ESM 1 (DOCX 56 kb)

## Data Availability

The data that support the findings of this study are available on request from the corresponding author.
