# Supplementary material for: Effect of short-term exposure to air pollution on daily cardio- and cerebrovascular hospitalisations in areas with a low level of air pollution
Source: Environ Sci Pollut Res Int. 2023 Sep 5;30(46):102438–45. doi: 10.1007/s11356-023-29544-z (PMC10567850; doi:10.1007/s11356-023-29544-z)
Supplement: Supplementary file 1 — (DOCX 56 kb) [file 11356_2023_29544_MOESM1_ESM.docx]

**Supplement Table 1:** Lag length selection using AIC, HQIC and SBIC.

| Lag Days | AIC | HQIC | SBIC |
| --- | --- | --- | --- |
| 0 | 16.0156 | 16.0395 | 16.0764 |
| 1 | 13.2092 | 13.4002*** | 13.6953*** |
| 2 | 13.1846 | 13.5429 | 14.0962 |
| 3 | 13.1926 | 13.718 | 14.5295 |
| 4 | 13.1903 | 13.883 | 14.9527 |
| 5 | 13.1478*** | 14.0077 | 15.3356 |
| 6 | 13.1933 | 14.2204 | 15.8065 |
| 7 | 13.2555 | 14.4498 | 16.2941 |
| 8 | 13.3035 | 14.665 | 16.7675 |
| 9 | 13.3566 | 14.8853 | 17.246 |
| 10 | 13.3971 | 15.093 | 17.7119 |

***denotes the lowest value

**Supplement Table 2:** Basic characteristics of disease admissions, meteorological data, and air pollution data.

| Variable | Mean (SD) | Min | Percentile | | | Max | IQR | Total |
| --- | --- | --- | --- | --- | --- | --- | --- | --- |
|  |  |  | 25^th^ | 50^th^ | 75^th^ |  |  |  |
| Daily Case Numbers*   - Cardiovascular and Cerebrovascular Disease (I20-I99) - Cardiovascular Disease (I20-I52; I70-I99) - Ischaemic Heart Disease (I20-I25) - Cerebrovascular Disease (I60-I69) - Haemorrhagic Stroke (I60-I62) - Ischaemic Stroke (I63) | 6.67 (2.57)  4.23 (2.07)  3.39 (1.83)  1.92 (1.41)  0.44 (0.68)  1.27 (1.15) | 0  0  0  0  0  0 | 5  3  2  1  0  0 | 6  4  3  2  0  1 | 8  5  5  3  1  2 | 18  13  10  8  4  6 | -  -  -  -  -  - | 4861  3344  2678  1517  351  999 |
| Meteorological   - Daily Maximum Temperature, °C - Total Daily Rainfall, mm | 24.83 (5.34)  2.50 (7.72) | 13.76  0 | 20.48  0 | 24.55  0.03 | 28.49  0.98 | 41.95  95.28 | 8.01  0.98 | -  - |
| Pollutant   - Sulphur Dioxide, SO_2_, pphm - Nitrogen Dioxide, NO_2_, pphm - Ozone, O_3_, pphm - Carbon Monoxide, CO, ppm - Ammonia, NH_3_, pphm - Particulate Matter, PM_10_, µg/m^3^ - Particulate Matter, PM_2.5_, µg/m^3^ | 0.18 (0.11)  0.73 (0.45)  1.79 (0.64)  0.25 (0.11)  1.19 (1.42)  29.09 (16.21)  10.05 (8.15) | 0.00  0.00  0.07  0.00  0.00  6.15  2.62 | 0.1  0.37  1.33  0.2  0.3  19.88  6.17 | 0.17  0.62  1.77  0.2  0.5  26.1  8.18 | 0.25  1.05  2.2  0.3  1.7  33.5  11.42 | 0.55  2.14  4.2  1.5  9.7  153.17  99.23 | 0.15  0.68  0.87  0.1  1.4  13.62  5.25 | -  -  -  -  -  -  - |

**Supplementary Table 3:** Spearman's rank coefficients between daily mean air pollutants and meteorological variables.

| Variable | SO_2_ | NO_2_ | O_3_ | CO | NH_3_ | PM_10_ | PM_2.5_ | Temperature | Rainfall |
| --- | --- | --- | --- | --- | --- | --- | --- | --- | --- |
| SO_2_ | 1.0000 |  |  |  |  |  |  |  |  |
| NO_2_ | 0.6340* | 1.0000 |  |  |  |  |  |  |  |
| O_3_ | -0.3388* | -0.4773* | 1.0000 |  |  |  |  |  |  |
| CO | 0.3460* | 0.2163* | -0.2239* | 1.0000 |  |  |  |  |  |
| NH_3_ | 0.4509* | 0.6931* | -0.3447* | 0.2514* | 1.0000 |  |  |  |  |
| PM_10_ | 0.0410 | -0.0828* | 0.3042* | 0.2416* | -0.0046 | 1.0000 |  |  |  |
| PM_2.5_ | 0.2365* | 0.2473* | 0.0773* | 0.4587* | 0.2497* | 0.7564* | 1.0000 |  |  |
| Temperature | -0.0534* | -0.3436* | 0.3339* | 0.2467* | -0.3016* | 0.6064* | 0.3266* | 1.0000 |  |
| Rainfall | -0.1408* | -0.1099* | -0.0193* | -0.0754* | -0.2124* | -0.4536* | -0.3585* | -0.2748 | 1.0000 |

*p-value <0.05 considered as significant

**Supplement Table 4:** Cardio and cerebrovascular hospitalisations' IRRs (95% CI) for every unit increase in ambient gaseous air pollutants in the single-pollutant model, across lag 0–5 days.

|  |  | IRR; 95% CI; p-value | | | | | | | | | | | | | | |
| --- | --- | --- | --- | --- | --- | --- | --- | --- | --- | --- | --- | --- | --- | --- | --- | --- |
| Outcome | **Lag** | **SO_2_** | | | **NO_2_** | | | **O_3_** | | | **CO** | | | **NH_3_** | | |
| Cardio and Cerebrovascular Disease | **0** | 1.29 | 0.98-1.68 | 0.07 | 1.12 | 1.03-1.22 | 0.01* | 0.94 | 0.89-0.98 | 0.01* | 1.26 | 0.98-1.62 | 0.06 | 1.00 | 0.98-1.02 | 0.79 |
|  | **1** | 1.41 | 1.02-1.96 | 0.04* | 1.13 | 1.03-1.23 | 0.01* | 0.94 | 0.89-0.99 | 0.02* | 1.30 | 1.00-1.70 | 0.05 | 1.00 | 0.98-1.02 | 0.99 |
|  | **2** | 1.66 | 1.14-2.41 | 0.01* | 1.13 | 1.02-1.25 | 0.02* | 0.94 | 0.89-1.00 | 0.04* | 1.32 | 1.00-1.76 | 0.05 | 1.00 | 0.97-1.02 | 0.74 |
|  | **3** | 1.77 | 1.18-2.65 | 0.01* | 1.12 | 1.01-1.25 | 0.04* | 0.95 | 0.89-1.01 | 0.08 | 1.30 | 0.95-1.78 | 0.10 | 0.99 | 0.96-1.01 | 0.50 |
|  | **4** | 1.66 | 1.08-2.56 | 0.02* | 1.08 | 0.96-1.20 | 0.21 | 0.96 | 0.90-1.02 | 0.14 | 1.23 | 0.89-1.71 | 0.21 | 0.98 | 0.95-1.01 | 0.21 |
|  | **5** | 1.55 | 0.98-2.43 | 0.06 | 1.07 | 0.95-1.20 | 0.27 | 0.96 | 0.90-1.02 | 0.15 | 1.26 | 0.89-1.77 | 0.19 | 0.98 | 0.95-1.01 | 0.25 |
| Cardiovascular Disease | **0** | 1.06 | 0.75-1.48 | 0.75 | 1.05 | 0.95-1.16 | 0.30 | 0.96 | 0.91-1.02 | 0.17 | 1.26 | 0.96-1.64 | 0.10 | 0.99 | 0.96-1.02 | 0.46 |
|  | **1** | 0.99 | 0.65-1.50 | 0.97 | 1.05 | 0.94-1.17 | 0.39 | 0.96 | 0.90-1.02 | 0.22 | 1.26 | 0.89-1.78 | 0.19 | 0.99 | 0.96-1.02 | 0.44 |
|  | **2** | 1.14 | 0.72-1.79 | 0.58 | 1.06 | 0.94-1.20 | 0.36 | 0.96 | 0.90-1.03 | 0.30 | 1.37 | 0.93-2.01 | 0.11 | 0.99 | 0.95-1.02 | 0.41 |
|  | **3** | 1.37 | 0.83-2.28 | 0.22 | 1.08 | 0.94-1.23 | 0.28 | 0.97 | 0.90-1.05 | 0.44 | 1.38 | 0.92-2.07 | 0.12 | 0.98 | 0.94-1.02 | 0.31 |
|  | **4** | 1.30 | 0.75-2.25 | 0.36 | 1.04 | 0.90-1.20 | 0.58 | 0.98 | 0.90-1.06 | 0.55 | 1.25 | 0.82-1.92 | 0.30 | 0.97 | 0.93-1.01 | 0.19 |
|  | **5** | 1.17 | 0.65-2.09 | 0.60 | 1.03 | 0.89-1.20 | 0.66 | 0.97 | 0.90-1.06 | 0.51 | 1.18 | 0.75-1.85 | 0.47 | 0.97 | 0.93-1.01 | 0.24 |
| Ischaemic Heart Disease | **0** | 1.13 | 0.77-1.63 | 0.54 | 1.08 | 0.97-1.21 | 0.17 | 0.97 | 0.91-1.03 | 0.30 | 1.31 | 0.97-1.79 | 0.08 | 0.99 | 0.95-1.02 | 0.42 |
|  | **1** | 1.02 | 0.64-1.63 | 0.93 | 1.07 | 0.94-1.21 | 0.29 | 0.97 | 0.90-1.04 | 0.42 | 1.31 | 0.91-1.90 | 0.15 | 0.98 | 0.95-1.02 | 0.41 |
|  | **2** | 1.11 | 0.66-1.86 | 0.71 | 1.07 | 0.93-1.23 | 0.34 | 0.98 | 0.90-1.06 | 0.57 | 1.45 | 0.96-2.21 | 0.08 | 0.98 | 0.94-1.02 | 0.34 |
|  | **3** | 1.36 | 0.76-2.41 | 0.30 | 1.09 | 0.93-1.27 | 0.29 | 0.98 | 0.90-1.07 | 0.63 | 1.49 | 0.98-2.31 | 0.07 | 0.97 | 0.94-1.02 | 0.34 |
|  | **4** | 1.31 | 0.70-2.43 | 0.40 | 1.06 | 0.90-1.25 | 0.48 | 0.98 | 0.90-1.07 | 0.68 | 1.36 | 0.86-2.15 | 0.19 | 0.98 | 0.93-1.01 | 0.34 |
|  | **5** | 1.17 | 0.61-2.56 | 0.64 | 1.05 | 0.88-1.25 | 0.58 | 0.97 | 0.89-1.06 | 0.56 | 1.30 | 0.80-2.13 | 0.29 | 0.98 | 0.93-1.01 | 0.38 |
| Cerebrovascular Disease | **0** | 1.66 | 1.00-2.73 | 0.05* | 1.26 | 1.09-1.45 | 0.00* | 0.90 | 0.83-0.99 | 0.02* | 1.08 | 0.69-1.69 | 0.74 | 1.01 | 0.97-1.05 | 0.73 |
|  | **1** | 2.27 | 1.20-4.28 | 0.01* | 1.27 | 1.08-1.50 | 0.00* | 0.91 | 0.83-1.01 | 0.07 | 1.30 | 0.77-2.17 | 0.33 | 1.01 | 0.97-1.07 | 0.57 |
|  | **2** | 2.81 | 1.36-5.81 | 0.01* | 1.28 | 1.06-1.54 | 0.01* | 0.92 | 0.83-1.03 | 0.14 | 1.24 | 0.69-2.23 | 0.479 | 1.01 | 0.95-1.07 | 0.75 |
|  | **3** | 2.46 | 1.09-5.56 | 0.03* | 1.22 | 0.99-1.49 | 0.06 | 0.93 | 0.83-1.04 | 0.19 | 1.22 | 0.65-2.29 | 0.546 | 0.99 | 0.93-1.06 | 0.83 |
|  | **4** | 2.23 | 0.94-5.32 | 0.07 | 1.18 | 0.94-1.47 | 0.16 | 0.94 | 0.83-1.05 | 0.28 | 1.26 | 0.67-2.41 | 0.479 | 0.98 | 0.92-1.05 | 0.63 |
|  | **5** | 2.23 | 0.90-5.53 | 0.08 | 1.20 | 0.95-1.52 | 0.12 | 0.94 | 0.83-1.06 | 0.30 | 1.56 | 0.79-3.07 | 0.196 | 0.99 | 0.92-1.06 | 0.67 |
| Haemorrhagic Stroke | **0** | 0.91 | 0.32-2.58 | 0.86 | 1.26 | 0.94-1.69 | 0.13 | 0.87 | 0.72-1.04 | 0.12 | 1.06 | 0.42-2.65 | 0.902 | 0.99 | 0.91-1.09 | 0.90 |
|  | **1** | 1.55 | 0.44-5.44 | 0.50 | 1.25 | 0.90-1.74 | 0.19 | 0.83 | 0.68-1.01 | 0.06 | 1.60 | 0.64-4.03 | 0.318 | 1.00 | 0.89-1.12 | 0.99 |
|  | **2** | 2.48 | 0.59-10.39 | 0.21 | 1.27 | 0.87-1.84 | 0.22 | 0.83 | 0.67-1.03 | 0.09 | 2.13 | 0.77-5.91 | 0.147 | 0.99 | 0.88-1.13 | 0.94 |
|  | **3** | 2.97 | 0.59-15.07 | 0.19 | 1.21 | 0.80-1.83 | 0.36 | 0.83 | 0.66-1.03 | 0.09 | 2.61 | 0.85-8.04 | 0.095 | 0.98 | 0.86-1.12 | 0.81 |
|  | **4** | 3.72 | 0.63-22.04 | 0.15 | 1.20 | 0.76-1.89 | 0.44 | 0.83 | 0.65-1.04 | 0.11 | 2.34 | 0.69-7.91 | 0.173 | 0.97 | 0.85-1.12 | 0.72 |
|  | **5** | 4.39 | 0.63-30.63 | 0.14 | 1.16 | 0.71-1.88 | 0.56 | 0.81 | 0.64-1.04 | 0.10 | 2.22 | 0.63-7.82 | 0.213 | 0.97 | 0.85-1.12 | 0.71 |
| Ischaemic Stroke | **0** | 1.94 | 1.09-3.44 | 0.02* | 1.27 | 1.06-1.52 | 0.01* | 0.93 | 0.84-1.04 | 0.19 | 0.96 | 0.55-1.69 | 0.884 | 1.01 | 0.95-1.07 | 0.81 |
|  | **1** | 2.67 | 1.21-5.89 | 0.02* | 1.31 | 1.06-1.62 | 0.01* | 0.96 | 0.85-1.08 | 0.52 | 1.09 | 0.57-2.09 | 0.748 | 1.01 | 0.94-1.07 | 0.86 |
|  | **2** | 3.10 | 1.24-7.76 | 0.02* | 1.33 | 1.05-1.68 | 0.02* | 0.98 | 0.86-1.12 | 0.80 | 0.94 | 0.44-1.98 | 0.862 | 1.01 | 0.94-1.08 | 0.84 |
|  | **3** | 2.57 | 0.92-7.17 | 0.07 | 1.29 | 0.99-1.68 | 0.06 | 0.99 | 0.86-1.14 | 0.86 | 0.86 | 0.38-1.95 | 0.726 | 0.99 | 0.92-1.06 | 0.85 |
|  | **4** | 2.09 | 0.71-6.24 | 0.18 | 1.23 | 0.92-1.64 | 0.17 | 1.00 | 0.86-1.16 | 1.00 | 0.95 | 0.42-2.18 | 0.906 | 0.98 | 0.90-1.06 | 0.54 |
|  | **5** | 1.94 | 0.62-6.03 | 0.26 | 1.27 | 0.94-1.73 | 0.12 | 1.01 | 0.86-1.18 | 0.92 | 1.27 | 0.52-3.08 | 0.527 | 0.97 | 0.90-1.05 | 0.49 |

*p-value <0.05 considered as significant

**Supplement Table 5:** Cardio and cerebrovascular hospitalisations' IRRs (95% CI) for every unit increase in ambient particulate matters in the single-pollutant model, across lag 0–5 days.

|  |  | IRR; 95% CI; p-value | | | | | |
| --- | --- | --- | --- | --- | --- | --- | --- |
| Outcome | **Lag** | **PM_10_** | | | **PM_2.5_** | | |
| Cardio and Cerebrovascular Disease | **0** | 1.00 | 1.00-1.00 | 0.901 | 1.00 | 1.00-1.00 | 0.781 |
|  | **1** | 1.00 | 1.00-1.00 | 0.613 | 1.00 | 1.00-1.00 | 0.988 |
|  | **2** | 1.00 | 1.00-1.00 | 0.908 | 1.00 | 1.00-1.00 | 0.893 |
|  | **3** | 1.00 | 1.00-1.00 | 0.965 | 1.00 | 1.00-1.00 | 0.899 |
|  | **4** | 1.00 | 1.00-1.00 | 0.964 | 1.00 | 1.00-1.00 | 0.764 |
|  | **5** | 1.00 | 1.00-1.00 | 0.872 | 1.00 | 1.00-1.00 | 0.753 |
| Cardiovascular Disease | **0** | 1.00 | 1.00-1.00 | 0.455 | 1.00 | 1.00-1.00 | 0.677 |
|  | **1** | 1.00 | 1.00-1.00 | 0.914 | 1.00 | 1.00-1.00 | 0.860 |
|  | **2** | 1.00 | 1.00-1.00 | 0.737 | 1.00 | 1.00-1.00 | 0.989 |
|  | **3** | 1.00 | 1.00-1.00 | 0.980 | 1.00 | 1.00-1.01 | 0.709 |
|  | **4** | 1.00 | 1.00-1.00 | 0.928 | 1.00 | 1.00-1.01 | 0.761 |
|  | **5** | 1.00 | 1.00-1.00 | 0.830 | 1.00 | 1.00-1.01 | 0.864 |
| Ischaemic Heart Disease | **0** | 1.00 | 1.00-1.00 | 0.243 | 1.00 | 0.99-1.00 | 0.517 |
|  | **1** | 1.00 | 1.00-1.00 | 0.567 | 1.00 | 0.99-1.00 | 0.593 |
|  | **2** | 1.00 | 1.00-1.00 | 0.350 | 1.00 | 0.99-1.00 | 0.728 |
|  | **3** | 1.00 | 1.00-1.00 | 0.624 | 1.00 | 1.00-1.01 | 0.876 |
|  | **4** | 1.00 | 1.00-1.00 | 0.705 | 1.00 | 1.00-1.01 | 0.827 |
|  | **5** | 1.00 | 1.00-1.00 | 0.849 | 1.00 | 0.99-1.01 | 0.878 |
| Cerebrovascular Disease | **0** | 1.00 | 1.00-1.00 | 0.768 | 1.00 | 0.99-1.00 | 0.599 |
|  | **1** | 1.00 | 1.00-1.01 | 0.487 | 1.00 | 0.99-1.01 | 0.978 |
|  | **2** | 1.00 | 1.00-1.01 | 0.781 | 1.00 | 0.99-1.01 | 0.988 |
|  | **3** | 1.00 | 1.00-1.01 | 0.791 | 1.00 | 0.99-1.01 | 0.831 |
|  | **4** | 1.00 | 1.00-1.01 | 0.634 | 1.00 | 0.99-1.01 | 0.881 |
|  | **5** | 1.00 | 1.00-1.01 | 0.559 | 1.00 | 0.99-1.01 | 0.873 |
| Haemorrhagic Stroke | **0** | 1.00 | 0.99-1.00 | 0.856 | 0.99 | 0.98-1.01 | 0.379 |
|  | **1** | 1.00 | 0.99-1.01 | 0.887 | 0.99 | 0.98-1.01 | 0.427 |
|  | **2** | 1.00 | 0.98-1.01 | 0.641 | 1.00 | 0.98-1.01 | 0.538 |
|  | **3** | 1.00 | 0.98-1.01 | 0.574 | 1.00 | 0.98-1.01 | 0.705 |
|  | **4** | 1.00 | 0.98-1.01 | 0.588 | 1.00 | 0.98-1.01 | 0.688 |
|  | **5** | 1.00 | 0.98-1.01 | 0.610 | 1.00 | 0.98-1.01 | 0.589 |
| Ischaemic Stroke | **0** | 1.00 | 1.00-1.00 | 0.951 | 1.00 | 0.99-1.01 | 0.927 |
|  | **1** | 1.00 | 1.00-1.01 | 0.327 | 1.00 | 1.00-1.01 | 0.471 |
|  | **2** | 1.00 | 1.00-1.01 | 0.466 | 1.00 | 0.99-1.01 | 0.554 |
|  | **3** | 1.00 | 1.00-1.01 | 0.476 | 1.00 | 0.99-1.01 | 0.838 |
|  | **4** | 1.00 | 1.00-1.01 | 0.379 | 1.00 | 0.99-1.01 | 0.802 |
|  | **5** | 1.00 | 1.00-1.01 | 0.279 | 1.00 | 0.99-1.01 | 0.524 |

*p-value <0.05 considered as significant

**Supplement Table 6:** Cardio and cerebrovascular hospitalisations' IRRs (95% CI) for every unit increase in ambient SO_2,_ NO_2,_ and O_3_ in the bi-pollutant model, across lag 0–5 days.

| Outcome | Lag | IRR (95% CI); p-value | | | | | |
| --- | --- | --- | --- | --- | --- | --- | --- |
|  |  | **Model 1: SO_2_ and NO_2_** | | **Model 2: SO_2_ and O_3_** | | **Model 3: NO_2_ and O_3_** | |
|  |  | **SO_2_** | **NO_2_** | **SO_2_** | **O_3_** | **NO_2_** | **O_3_** |
| Cardio and Cerebrovascular Disease | 0 | 1.07 (0.77-1.47); 0.694 | **1.11 (1.01-1.22); 0.034***** | 1.17 (0.89-1.55); 0.258 | **0.94 (0.90-0.99); 0.016***** | 1.09 (1.00-1.19); 0.052 | 0.96 (0.91-1.00); 0.067 |
|  | 1 | 1.16 (0.78-1.74); 0.456 | 1.10 (0.99-1.23); 0.090 | 1.28 (0.91-1.80); 0.150 | 0.95 (0.90-1.00); 0.060 | 1.09 (0.99-1.20); 0.063 | 0.96 (0.91-1.01); 0.123 |
|  | 2 | 1.44 (0.91-2.27); 0.116 | 1.07 (0.95-2.21); 0.274 | **1.54 (1.04-2.26); 0.030***** | 0.96 (0.91-1.02); 0.183 | 1.10 (0.99-1.23); 0.067 | 0.96 (0.91-1.02); 0.201 |
|  | 3 | 1.61 (0.99-2.64); 0.055 | 1.04 (0.91-1.19); 0.529 | **1.66 (1.09-2.53); 0.019***** | 0.97 (0.91-1.03); 0.313 | 1.10 (0.98-1.23); 0.114 | 0.96 (0.91-1.03); 0.258 |
|  | 4 | 1.68 (1.00-2.82); 0.052 | 0.99 (0.87-1.14); 0.935 | 1.57 (1.00-2.46); 0.049 | 0.97 (0.92-1.04); 0.409 | 1.05 (0.93-1.18); 0.411 | 0.96 (0.91-1.03); 0.258 |
|  | 5 | 1.54 (0.90-2.65); 0.115 | 1.00 (0.87-1.15); 0.997 | 1.45 (0.90-2.32); 0.123 | 0.97 (0.91-1.03); 0.358 | 1.04 (0.92-1.18); 0.503 | 0.96 (0.90-1.03); 0.245 |
| Cerebrovascular Disease | 0 | 1.13 (0.62-2.08); 0.694 | **1.24 (1.04-1.47); 0.015***** | 1.47 (0.87-2.46); 0.147 | 0.92 (0.85-1.01); 0.073 | **1.22 (1.05-1.42); 0.009***** | 0.95 (0.87-1.04); 0.260 |
|  | 1 | 1.62 (0.74-3.53); 0.229 | 1.18 (0.97-1.44); 0.103 | **2.05 (1.06-3.96); 0.032***** | 0.95 (0.86-1.05); 0.281 | **1.24 (1.04-1.48); 0.017***** | 0.96 (0.86-1.06); 0.426 |
|  | 2 | 2.16 (0.88-5.26); 0.091 | 1.14 (0.91-1.43); 0.271 | **2.62 (1.22-5.60); 0.013***** | 0.96 (0.86-1.08); 0.517 | **1.25 (1.02-1.53); 0.030***** | 0.96 (0.86-1.08); 0.542 |
|  | 3 | 2.05 (0.74-5.63); 0.165 | 1.09 (0.84-1.41); 0.508 | 2.26 (0.96-5.31); 0.061 | 0.96 (0.85-1.08); 0.495 | 1.18 (0.95-1.48); 0.141 | 0.96 (0.84-1.08); 0.468 |
|  | 4 | 1.97 (0.67-5.77); 0.216 | 1.06 (0.80-1.40); 0.677 | 2.07 (0.84-5.11); 0.112 | 0.97 (0.85-1.09); 0.575 | 1.15 (0.90-1.46); 0.266 | 0.96 (0.84-1.09); 0.513 |
|  | 5 | 1.83 (0.60-5.57); 0.289 | 1.10 (0.82-1.47); 0.519 | 2.06 (0.80-5.29); 0.133 | 0.96 (0.85-1.10); 0.586 | 1.17 (0.92-1.51); 0.203 | 0.96 (0.84-1.10); 0.556 |
| Cerebral Infarction | 0 | 1.38 (0.67-2.83); 0.377 | 1.21 (0.97-1.51); 0.095 | 1.83 (1.00-3.37); 0.051 | 0.96 (0.86-1.07); 0.492 | **1.26 (1.04-1.54); 0.019***** | 0.98 (0.88-1.10); 0.785 |
|  | 1 | 1.87 (0.71-4.93); 0.208 | 1.19 (0.92-1.56); 0.181 | **2.73 (1.19-6.23); 0.018***** | 1.01 (0.89-1.14); 0.871 | **1.33 (1.06-1.67); 0.013***** | 1.02 (0.90-1.17); 0.723 |
|  | 2 | 2.21 (0.73-6.62); 0.159 | 1.18 (0.89-1.56); 0.258 | **3.33 (1.27-8.76); 0.015***** | 1.04 (0.90-1.20); 0.611 | **1.36 (1.05-1.76); 0.018***** | 1.04 (0.90-1.21); 0.566 |
|  | 3 | 1.83 (0.54-6.19); 0.331 | 1.18 (0.82-1.62); 314 | 2.74 (0.94-8.04); 0.066 | 1.03 (0.89-1.20); 0.686 | 1.32 (0.99-1.75); 0.059 | 1.04 (0.89-1.22); 0.634 |
|  | 4 | 1.56 (0.43-5.66); 0.495 | 1.15 (0.82-1.62); 0.429 | 2.26 (0.73-7.03); 0.160 | 1.04 (0.89-1.21); 0.665 | 1.26 (0.92-1.71); 0.146 | 1.04 (0.89-1.22); 0.629 |
|  | 5 | 1.25 (0.33-4.65); 0.743 | 1.23 (0.86-1.76); 0.246 | 2.11 (0.65-6.89); 0.215 | 1.04 (0.88-1.23); 0.634 | 1.32 (0.95-1.82); 0.096 | 1.05 (0.89-1.25); 0.534 |

***p-value <0.05 considered as significant
